# An End-To-End Pipeline for Fully Automatic Morphological Quantification of Mouse Brain Structures From MRI Imagery

**DOI:** 10.3389/fbinf.2022.865443

**Published:** 2022-06-08

**Authors:** Shahinur Alam, Tae-Yeon Eom, Jeffrey Steinberg, David Ackerman, J. Eric Schmitt, Walter J. Akers, Stanislav S. Zakharenko, Khaled Khairy

**Affiliations:** ^1^ Center for Bioimage Informatics, St. Jude Children’s Research Hospital, Memphis, TN, United States; ^2^ Department of Developmental Neurobiology, St. Jude Children’s Research Hospital, Memphis, TN, United States; ^3^ Center for in Vivo Imaging and Therapeutics, St. Jude Children’s Research Hospital, Memphis, TN, United States; ^4^ Scientific Computing, Janelia Research Campus, Ashburn, VA, United States; ^5^ Brain Behavior Laboratory, Departments of Psychiatry and Radiology, University of Pennsylvania, Philadelphia, PA, United States

**Keywords:** MRI, deep learning, image segmentation, image registration, brain atlas, data augmentation, mouse brain

## Abstract

Segmentation of mouse brain magnetic resonance images (MRI) based on anatomical and/or functional features is an important step towards morphogenetic brain structure characterization of murine models in neurobiological studies. State-of-the-art image segmentation methods register image volumes to standard presegmented templates or well-characterized highly detailed image atlases. Performance of these methods depends critically on the quality of skull-stripping, which is the digital removal of tissue signal exterior to the brain. This is, however, tedious to do manually and challenging to automate. Registration-based segmentation, in addition, performs poorly on small structures, low resolution images, weak signals, or faint boundaries, intrinsic to *in vivo* MRI scans. To address these issues, we developed an automated end-to-end pipeline called DeepBrainIPP (deep learning-based brain image processing pipeline) for 1) isolating brain volumes by stripping skull and tissue from T2w MRI images using an improved deep learning-based skull-stripping and data augmentation strategy, which enables segmentation of large brain regions by atlas or template registration, and 2) address segmentation of small brain structures, such as the paraflocculus, a small lobule of the cerebellum, for which DeepBrainIPP performs direct segmentation with a dedicated model, producing results superior to the skull-stripping/atlas-registration paradigm. We demonstrate our approach on data from both *in vivo* and *ex vivo* samples, using an in-house dataset of 172 images, expanded to 4,040 samples through data augmentation. Our skull stripping model produced an average Dice score of 0.96 and residual volume of 2.18%. This facilitated automatic registration of the skull-stripped brain to an atlas yielding an average cross-correlation of 0.98. For small brain structures, direct segmentation yielded an average Dice score of 0.89 and 5.32% residual volume error, well below the tolerance threshold for phenotype detection. Full pipeline execution is provided to non-expert users *via* a Web-based interface, which exposes analysis parameters, and is powered by a service that manages job submission, monitors job status and provides job history. Usability, reliability, and user experience of DeepBrainIPP was measured using the Customer Satisfaction Score (CSAT) and a modified PYTHEIA Scale, with a rating of excellent. DeepBrainIPP code, documentation and network weights are freely available to the research community.

## 1 Introduction

Segmentation of brain structures from magnetic resonance (MRI) images is an important step for accurate morphometric measurement and morphogenetic characterization and is a rate-limiting step for neuroimaging studies. Our work is motivated by a need to perform such segmentation automatically and robustly for large and small (less than about 5% by volume) mouse brain regions for hundreds of recordings of both *in vivo* and *ex vivo* MRI image volumes for both wild-type and mutants. In order to be accessible to research staff regardless of level of computational expertise, we also required our method to be easy to use.

Although manual segmentation of brain structures by domain-experts is considered to be accurate and reliable (Dong, 2008), it is labor intensive, time-consuming, and therefore impractical for evaluating the large datasets essential for evaluating small changes in brain structures resulting from interventions or genetic modifications. Automated methods have traditionally been limited by the number of brain structures that can be segmented (Tan et al., 2020), or have required multiple expert-annotated atlases (Ma et al., 2014) increasing study time and complicating the analysis. Such methods made use of hand-crafted features to segment brain regions (Clarke et al., 1995; Balafar et al., 2010; Nanthagopal and Sukanesh, 2013), which is not robust against variations in tissue geometry and imaging modalities.

Most recent advancements in machine learning, especially deep learning, have revolutionized segmentation of medical images. Deep learning-based methods, especially convolutional neural networks (CNN) (Krizhevsky et al., 2012), eliminate the burden of feature engineering and automatically extract robust features. In recent years, researchers have developed several high performing CNN model architectures for image segmentation (Long et al., 2015; Milletari et al., 2016; Chen et al., 2017, 2018; Badrinarayanan et al., 2017; Jégou et al., 2017; Ellis and Aizenberg, 2020; Hsu et al., 2020; Tan et al., 2020). The U-Net (Ronneberger et al., 2015), one such CNN-based architecture, is particularly widely used for medical image segmentation because of its high accuracy. It is an encoder-decoder based architecture consisting of a contracting and a symmetrically expanding path to capture context and produce precise localization.

Such advanced machine learning-based methods must ideally be trained on large ground truth datasets laboriously annotated by experts. This task is demanding for high-resolution volumetric images and large structures within them. Therefore, in addition to deep learning methods, researchers rely on a paradigm of automated image registration to an atlas. This approach does not require manual annotation beyond the original template or atlas. The accuracy of such registration-based segmentation, depends on the performance of a pre-registration skull-stripping step, in which skull tissue appearing in the image is digitally removed, isolating the brain tissue (Woods et al., 1993; Klein et al., 2010; Kleesiek et al., 2016). Skull stripping can benefit from improvements provided by modern deep learning methodology, because it is more accessible for initial manual generation of ground truth by expert annotation.

In recent years, several highly performant automated skull stripping tools have been developed for human MRI research (Cox, 1996; Shattuck and Leahy, 2002; Leung et al., 2011; Doshi et al., 2013; Isensee et al., 2019). Unfortunately, those tools are not well-suited for mouse brain skull-stripping from MRI data acquired at high magnetic fields. Moreover, human and mouse MRI images differ significantly in anatomical structure and tissue geometry, further reducing the efficacy of re-using those machine learning models. Therefore some recent works focused on MRI-derived mouse brain structure directly [MU-Net (De Feo et al., 2021), U-Net (Hsu et al., 2020), RATS (Oguz et al., 2014), PCNN (Chou et al., 2011), SHERM (Liu et al., 2020), (Schell et al., 2019)]. This is a fast developing field. To the best of our knowledge, the most recent mouse brain extraction models have been developed by De Feo et al. (2021) and Hsu et al. (2020). De Feo et al. (2021) developed a U-net like architecture (MU-Net) for both skull stripping and segmentation of brain regions. They demonstrate their results on segmentation of the cortex, hippocampi, striati and brain ventricles. Skull stripping and region segmentation are treated as separate tasks with two output maps generated from the final block of the U-net’s decoder branch. MU-Net was trained on T2w mouse brain MRI data with each brain delineated with a bounding box. To automate the task of bounding box generation, an auxiliary network is included in the MU-Net system. The skull stripping performance reported in that work produces a Dice score of 0.978 when trained on the datasets available to their study. However, a much lower Dice score of 0.577 (see Results and Discussion) was obtained when we used the same network weights with our dataset (T2w TSE). Hsu et al. (2020) based their work on a U-net architecture to perform 2D slice-by-slice segmentation. They reported a skull stripping Dice score of 0.85 on a T2w RARE dataset. Although this is a suitable performance level for many applications, improvement is necessary for more nuanced morphogenetic studies; especially those targeting both large and small brain regions.

To achieve such improvement, and develop highly performing segmentation models for both skull-stripping and targeted region segmentation, we took advantage of the U-net approach and its variant proposed by Isensee et al. (2017) motivated by its power to combine feature abstraction and localization.

Moreover, as stated above, expert neurobiologists require an integrated solution that performs skull stripping and image segmentation in a reliable and accessible manner at large scale, even without computational expertise. With this additional goal in mind we were motivated to develop DeepBrainIPP (deep learning-based brain image processing pipeline), which we present in this paper. DeepBrainIPP facilitates high-throughput brain structure segmentation. Objective evaluation of our neural network models and subjective evaluation of the end-to-end system demonstrate the high utility of the DeepBrainIPP approach.

The rest of this paper is organized as follows: in Section 2, we introduce the dataset, our computational approach, and describe the software. In Section 3, we present results of running our analysis and discuss performance, limitations and evaluation of our approach. We conclude in Section 4 with some remarks.

## 2 Dataset and Methods

### 2.1 Dataset Details

To develop our approach, and validate our machine learning models, we focused on two in-house-collected mouse brain MRI datasets (*in vivo* and *ex vivo* image volumes). *In vivo* MRI images are collected in anesthetized mice at a lower resolution, provide less detail and contrast compared to *ex vivo* images and are therefore harder to segment. This type of data is important for many studies, because image acquisition time is shorter so longitudinal data collection is possible. We were therefore motivated to include this type of analysis in our pipeline. Details of MRI image data are provided below.

#### 2.1.1 *In Vivo* MRI

Magnetic resonance imaging (MRI) was performed on a Bruker Clinscan 7T MRI system (Bruker Biospin MRI GmbH, Ettlingen, Germany). Prior to scanning, mice were anesthetized in a chamber (3% Isoflurane in oxygen delivered at 1 L/min) and maintained using nose-cone delivery (1–2% Isoflurane in oxygen delivered at 1 L/min). Animals were provided thermal support using a heated bed with warm water circulation and a physiological monitoring system to monitor breath rate. MRI was acquired with a 2-channel mouse brain surface receive coil positioned over the mouse head and placed inside a 72 mm transmit/receive coil. After the localizer, a T2-weighted turbo spin echo sequence was performed in the coronal (TR/TE = 2,500/42 ms, matrix size = 320 × 320, field of view = 25 mm × 25 mm, slice thickness = 0.5 mm, number of slices = 14), sagittal (TR/TE = 2,550/39 ms, matrix size = 320 × 320, field of view = 25 mm × 25 mm, slice thickness = 0.7 mm, number of slices = 16), and axial (TR/TE = 1910/42 ms, matrix size = 320 × 320, field of view = 25 mm × 25 mm, slice thickness = 0.6 mm, number of slices = 22, total time = 3 h 4 min coronal scan) orientations.

#### 2.1.2 *Ex Vivo* MRI

After a minimum of 24 h to fix the tissue, the brain was transferred to a 15 ml conical centrifuge tube and filled with Fomblin, a solution that is invisible to proton MRI. The tube is then placed in the MRI with a 2-channel mouse brain surface receive coil and a 72 mm volume transmit/receive coil. After the localizer, a T2-weighted 3D turbo spin echo sequence was performed in the coronal orientation (TR/TE = 1800/70 ms, matrix size = 252 × 384 × 176, echo train length = 9, averages = 6, total time = 16 h 7 min) with an isotropic resolution of 60 μm.

A total of 172 image volumes were collected and annotated by experts (*in vivo*: 147, *ex vivo*: 25). The quality of annotation was monitored by a separate group of experts not involved in the annotation procedure. Due to the small annotated sample size, we applied data augmentation techniques to train machine learning models that are robust against realistic changes in geometric and photometric properties (see section “Data augmentation”).

### 2.2 Summary of the DeepBrainIPP Workflow

**FIGURE 1:**
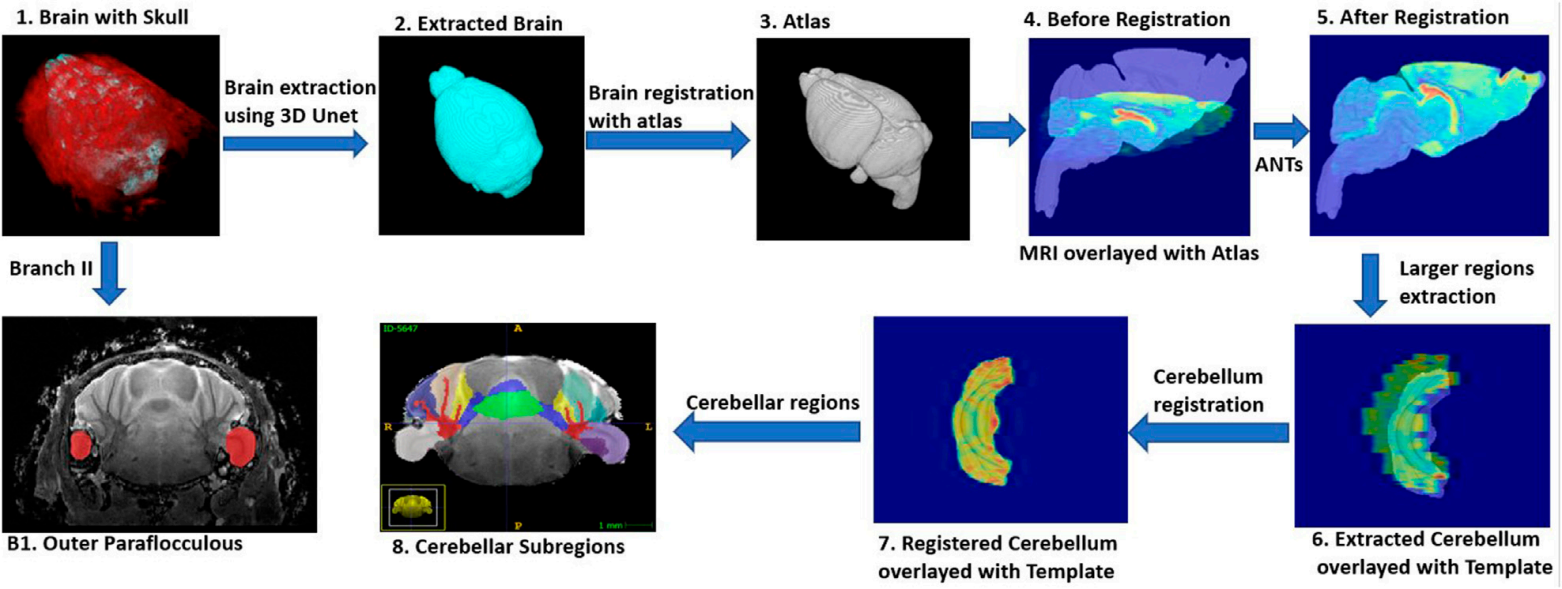
DeepBrainIPP workflow (Steps 1–5) skull stripping followed by registration of the segmented brain to an atlas (Step 6) Large brain structures are segmented (Steps 7–8) To segment sub-cerebellar regions (colors in Step 8), DeepBrainIPP registers the cerebellum to an in-house generated template. Step B1 following Branch II shows direct segmentation of the outer paraflocculus mask (red color) overlayed with the raw image. This direct segmentation outperformed the template registration approach in the case of small regions.

**FIGURE 2:**
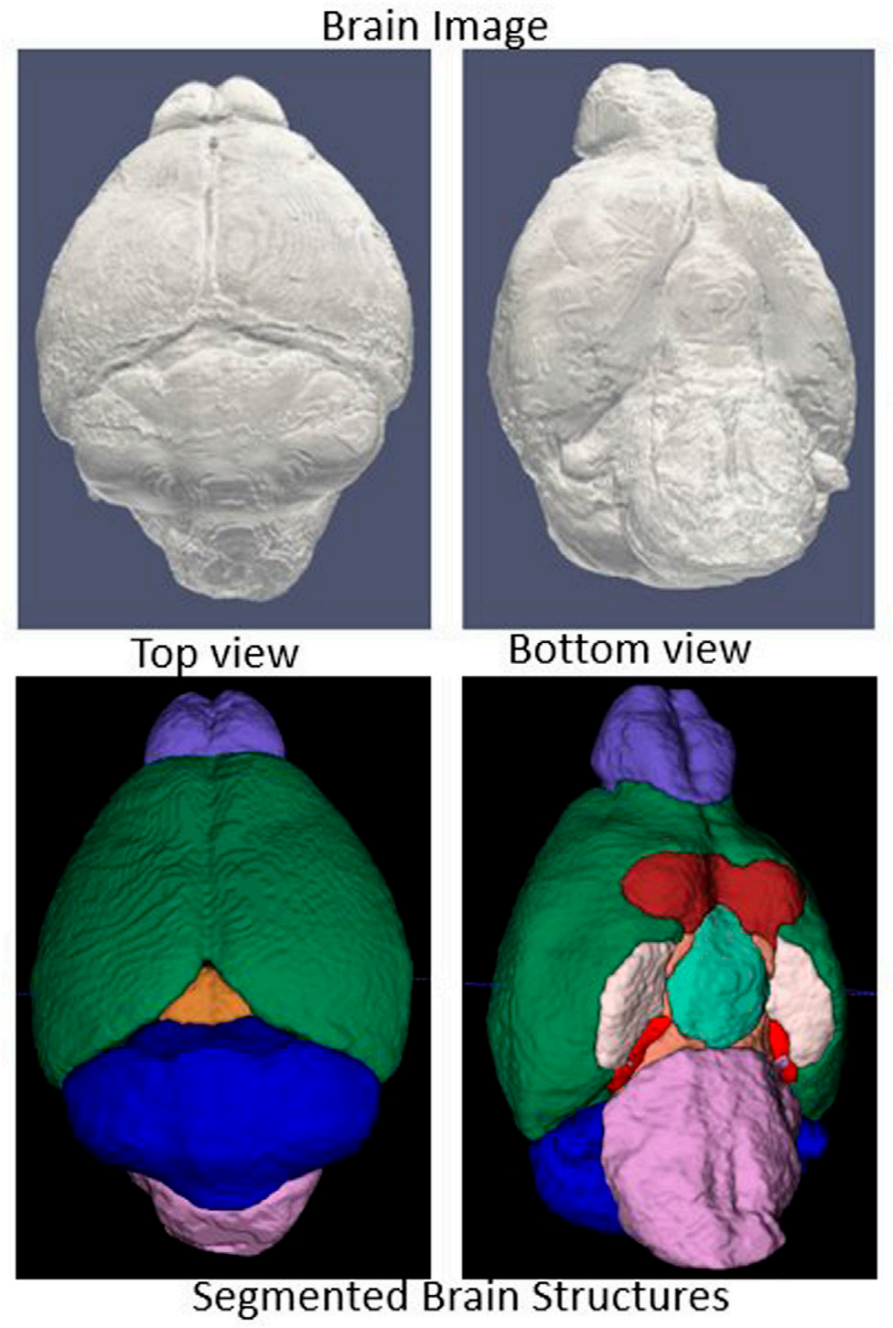
Segmentation outcomes: Top row: Segmented brain surface. Bottom row: Segmented large brain regions (blue color: cerebellum, Green color: fore brain, violet color: olfactory bulb, orchid color: brain stem).

The full workflow for segmenting brain structures is shown in Figure 1. First, we perform skull stripping for the whole brain using a 3D U-Net based model (see Model Development and Figure 1 steps 1–2). We then register the resulting segmented brain to an atlas or template (see Image Registration, and Figure 1 steps 3–6), and extract large brain structures by applying an inverse transformation to the atlas or template mask. A sample brain volume and segmented large brain structures are shown in Figure 2 (Optional) As a final step, the user can apply an additional registration-based segmentation to a detailed mask of a large brain region to further segment large-to-medium sized regions. As an example we show an application to the segmented cerebellum, which we register to an in-house generated template to further characterize its subregions (Figure 1 steps 6–8).

However, in cases where structures are small, or boundaries are weak or unclear (see Image Registration), the atlas-based registration does not perform well. Therefore, we developed a deep learning-based model that performs a direct segmentation for small regions such as the outer paraflocculus, a small lobule of the cerebellum, with the full raw data as its input (Figure 1 Branch II). In the case of such small regions, generation of ground truth data is significantly less laborious compared to large-region annotation.

A note on large vs. small region segmentation strategy: Manual annotation of MRI volumes for large regions at high precision and full resolution is not practical. This is the main motivation to use a skull-stripping/atlas-registration paradigm for large regions. In that paradigm, manual annotation of only one large region (which is the brain proper) one time per brain is needed. On the other hand, preparing training samples for small regions is significantly less laborious. For example, it takes up to 30 h for an expert to annotate the cerebellum from an *ex vivo* MRI image (an example for a large region). Whereas the same annotator can annotate the paraflocculus (a small region) within 20 min.

Finally, we perform morphological analysis on the extracted regions and generate summary reports for assessments. The measured volumes of extracted brain structures are used for phenotype detection. The complete workflow is available in the form of a web application with an accessible user interface that is intuitive to users without computational expertise (see Software).

### 2.3 Data Augmentation

Data augmentation is a strategy that artificially increases the size (and diversity of) a training dataset by applying realistic transformations. This strategy addresses two issues: 1) Model robustness: Automated brain image segmentation *via* deep learning requires large numbers of training samples with representative variability to perform robustly against changes in tissue geometry, contrast, tissue density, field of view, sample orientation, and imaging conditions (Halevy et al., 2009; Sun et al., 2017). It is not practically possible to acquire images covering all variability, or to annotate a sufficient number of such images. 2) Data augmentation significantly reduces overfitting of trained models (Shorten and Khoshgoftaar, 2019).

**FIGURE 3:**
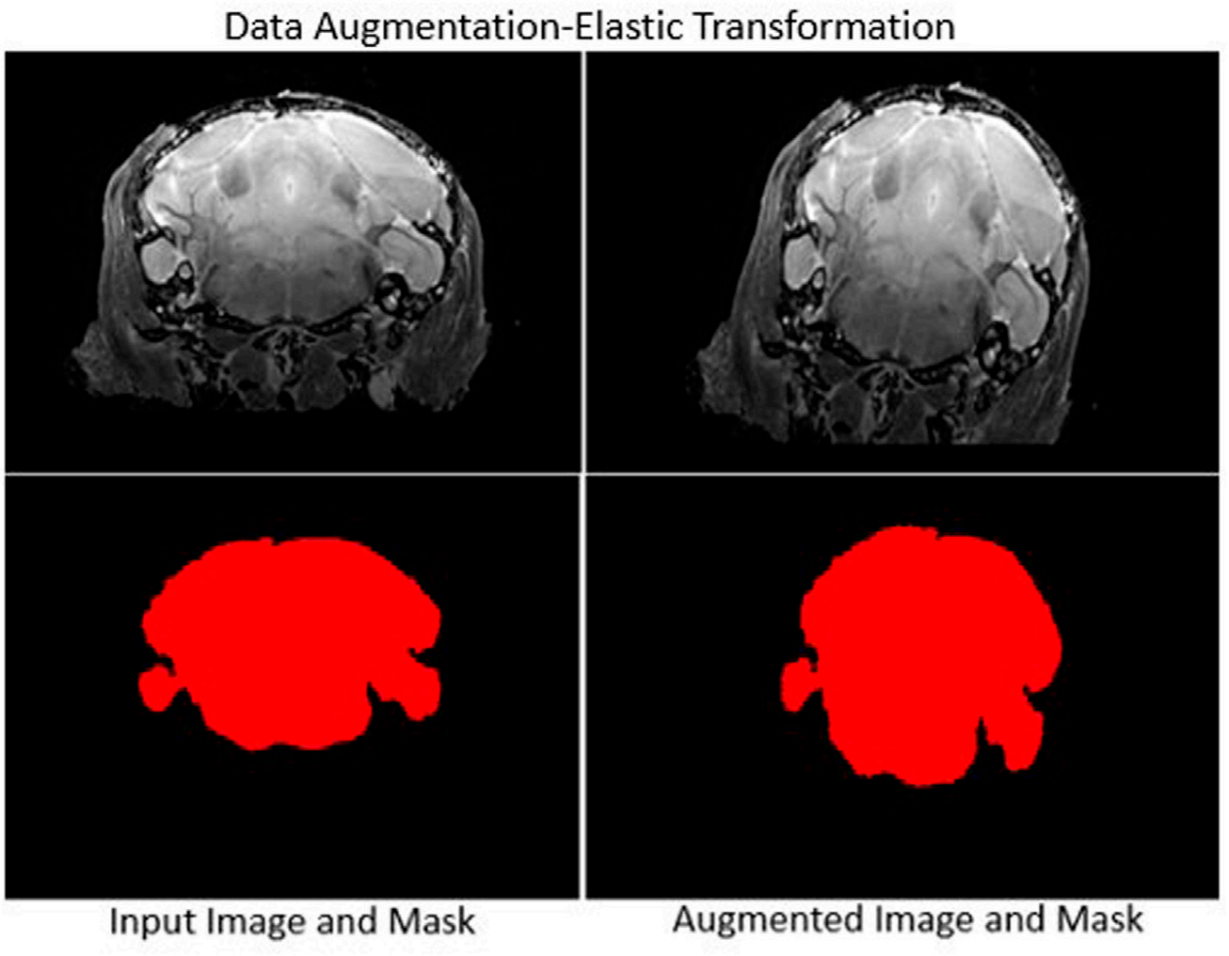
Sample data augmentation: Left column: Input image (top) and skull-stripped brain mask (bottom). Right column: Augmented transformed image and mask. Elastic deformation is shown. It provides model robustness against changes in tissue geometry.

Data augmentation requires careful selection of spatial transformations, intensity filters and associated parameters. We selected a set of transformation methods and parameters after extensive testing (see Table 1), and applied them to all images without combining transformations. A sample outcome of data augmentation with elastic transformation is shown in Figure 3.

**TABLE 1:** Data augmentation methods and parameters. Parameter values are chosen randomly from the sample space (square brackets).

Method’s name	Parameter value/Ranges	Augmentation nature
Horizontal Flip		Generates horizontally flipped images
Vertical Flip		Generates vertically flipped images
Dropout	(0.01, 0.05)	Generate images by dropping 1–5% voxels
Piecewise Affine	Scale (0.01, 0.07)	Generates images applying an affine transformation to a local grid
Elastic Transformation	alpha (2.5, 50), sigma (1,11)	Generates images by moving voxels locally
Additive Gaussian Noise	scale (0.0, 12.75)	Generates images by adding noise sampled from gaussian distributions
GaussianBlur	sigma (0.8, 1.5)	Generates smoothed images
Affine Transformation	rotation along *Z* axis(−20°,20°) scale (0.8,1.3) isotropic Translation (−0.05%, 0.05%)	Generates images by applying an affine transformation
Rotation	along *Y* axis [-20°,20°]	Generates images by rotating around the *Y* axis
CLAHE Zuiderveld, (1994)	Apply: yes/no	Generates contrast enhanced images

### 2.4 Deep Learning Models

#### 2.4.1 Network Architecture

**FIGURE 4:**
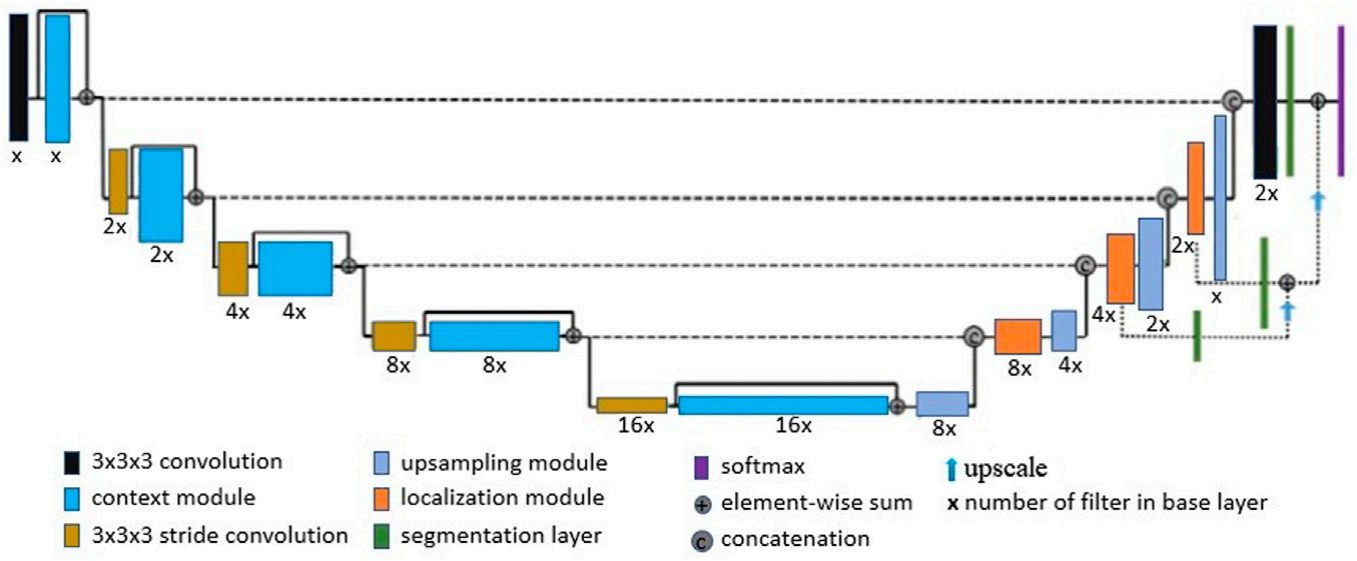
Network Architecture: We derived our network architecture from the work proposed by Isensee et al. (2017), in which 16 base layer filters and a depth of 5 were used. We treated both network depth and number of filters in the base layer as hyperparameters, which were determined by a Bayesian optimization and grid search.

For skull-stripping and small brain region segmentation (applied to the paraflocculus), we used a deep learning network architecture derived from the work of Isensee et al. (2017). Details of the network architecture are provided in Figure 4. Isensee et al. (2017) based their architecture on the U-net (Ronneberger et al., 2015) and variants proposed by Kayalibay et al. (2017), and applied it to brain tumor segmentation and radiomics survival prediction. In summary: the network’s context aggregation pathway extracts low-level features from input 3D images, encoding them into high-level abstractions. The network’s localization pathway then recombines higher level abstractions and lower level features for precise localization of the region of interest. Computation of activation maps in the context module is performed by a residual unit (He et al., 2016), which counteracts vanishing gradients and allows training of very deep networks. At each level (first half of the “U”) spatial resolution is reduced and the number of filters is increased. In the localization pathway, feature dimension is reduced and high level representations are up-scaled gradually to produce an output that has the same dimensions as the input. Isensee et al. (2017) used 16 filters in the base layer and five total levels. In our implementation, we treated the U-net depth and number of filters in the base layer as part of a set of hyperparameters, together with learning rate and dropout rate. To search for optimal hyperparameter values, we used a rough grid search followed by Bayesian optimization (Snoek et al., 2012), as implemented in the Keras Tuner (O’Malley et al., 2019) library. Moreover, we used instance normalization (Ulyanov et al., 2016) to prevent potential internal co-variance shift, in order to reduce training time. In addition, we addressed potential class imbalance issues (typically arising from an imbalanced number of background-voxels in MRI images relative to ROI voxels), by using a class-weighted Dice loss function.

#### 2.4.2 Data Processing

**FIGURE 5:**
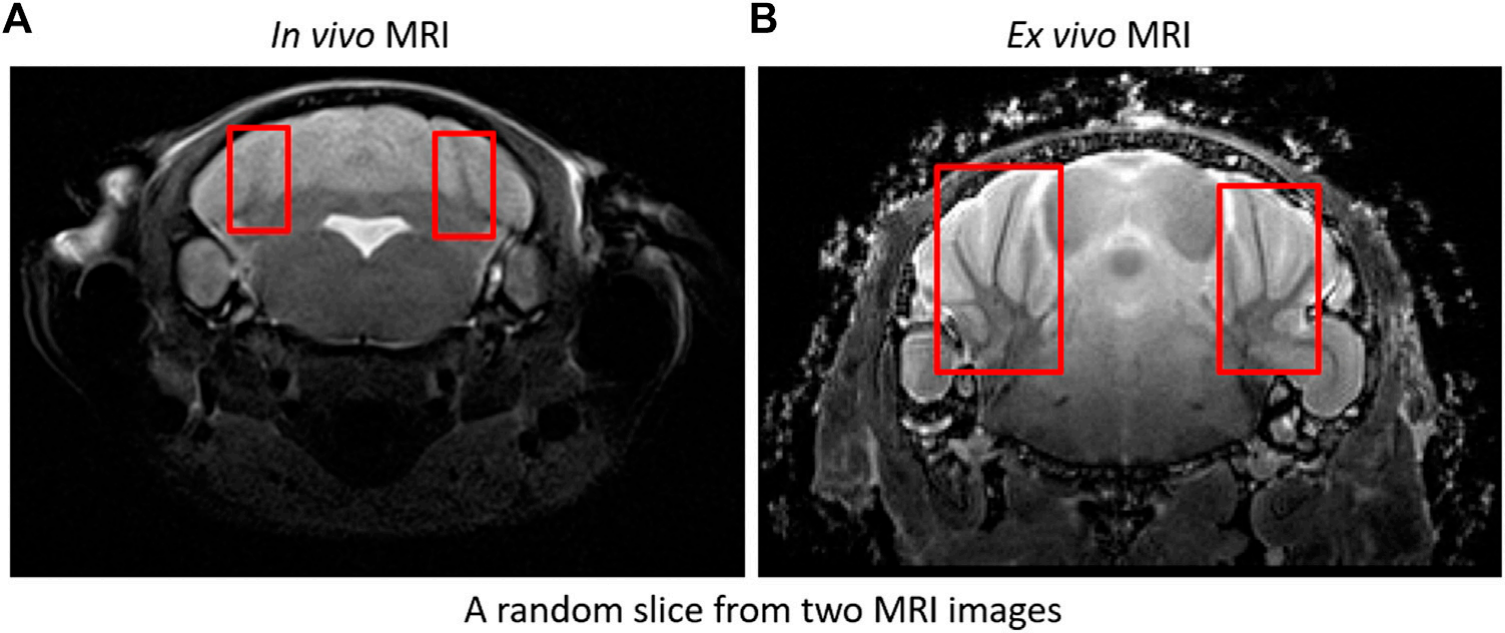
*In vivo* and *ex vivo* MRI images: Sample *in vivo* **(A)** and *ex vivo* **(B)** MRI images. *Ex vivo* recordings show significantly improved resolution and contrast, and are therefore easier to segment. For example: Branches (arbors vitae) are clearly visible in *ex vivo* compared to *in vivo*. See boxes.

We processed MRI imagery obtained for two protocols; *in vivo* and *ex vivo* imaging. Although same modality, data was obtained from two separate instruments resulting in differences in resolution, overall image spatial dimensions and contrast (see Figure 5). We developed separate deep learning models for each case, and datasets were resampled to 0.06 mm × 0.06 mm × 0.48 mm and 0.06 mm × 0.06 mm × 0.06 mm for training *in vivo* and *ex vivo* models respectively. We applied data augmentation as stated above to generate 4,040 training samples (from originally 172 image volumes) for skull stripping models and 1,500 samples (from originally 60 recorded image volumes) for the paraflocculus direct segmentation models. All training samples were cropped automatically based on the image center of mass and padded with zeros to obtain uniform spatial dimensions. Resampled dimensions of *in vivo* and ex-vio MRI images were 448 × 448 × 48 voxel^3^ and 256 × 224 × 288voxel^3^ respectively. We performed Z-score normalization on the training samples to increase model robustness against changes in intensity. Network models were trained with the whole image (rather than patches) to reduce training time and avoid tiling artifacts. This is one of the reasons why training DeepBrainIPP required large GPU memory (see “MODEL PARAMETERS AND MEMORY REQUIREMENTS” in Supplementary Material). Moreover, evaluating our model on an independent dataset, scores were calculated from the whole image.

#### 2.4.3 Skull Stripping Model Training

**FIGURE 6:**
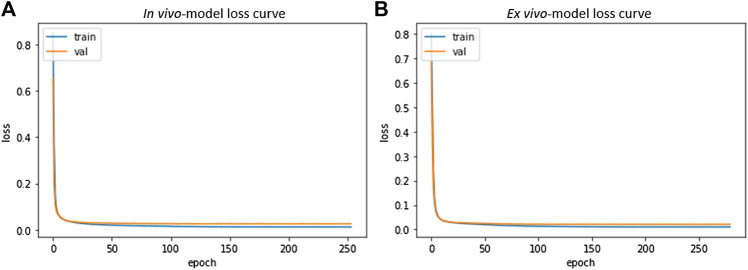
Skull Stripping Model-Loss curve. The smooth decay of validation loss for both *in vivo* **(A)** and *ex vivo* **(B)** demonstrate convergence of the learning process and a good fit for the resulting model.

*In vivo* and *ex vivo* skull-stripping models were trained on 4,040 samples of dimension 448 × 448 × 48 and 256 × 224 × 288 respectively for 500 epochs (with an early stopping if validation loss does not improve) using the Adam optimizer (Kingma and Ba, 2014). We used a batch size of 1, limited by available GPU memory (See“Model Parameters and Memory Requirements” in Supplementary Material). 80% of the dataset was used for training and 20% for validation. Optimal hyperparameters (depth of network, initial learning rate, dropout rate and number of filters in the base level) of *in vivo* and *ex vivo* models were 5, 5e-5, 0.1, 16 and 5, 5e-5, 0.2, 16 respectively. The learning rate was gradually reduced by a factor of 0.7 with a patience of 15 epochs if validation loss showed no improvement. The gradual reduction of learning rate reduces the step size as the global optimum is approached. This helps the network learn nuance from the data and potentially reduces the risk of overshooting beyond the global optimum. Calculations were performed on a compute node with four NVIDIA DGX A100 GPUs with 40 GB memory per GPU, over a period of 4 days to reach completion. Figure 6 shows the resulting loss curve in case of a skull stripping model. Results from this step are suitable for subsequent registration to an atlas for segmentation of large brain areas (see “Image Registration”).

#### 2.4.4 Small Region (Paraflocculus) Segmentation Model Training

Segmentation of small brain regions is more challenging and cannot always be addressed with the skull-stripping/atlas-registration paradigm. One example of such a region is the paraflocculus (see Figure 1 panel B1). The paraflocculus registers poorly to atlases with the global skull-stripping/atlas registration approach. Similar to our approach with skull-stripping, we built separate models for *in vivo* and *ex vivo* data, and models were trained on 1,500 samples of dimension 448 × 448 × 48 and 256 × 224 × 288 respectively with a batch size of one using the Adam optimizer for 500 epochs. Optimal hyperparameters for network depth, initial learning rate, dropout rate and number of base-level filter were obtained from a grid search followed by Bayesian optimization. The final set used for the *ex vivo* model: network depth 4, initial learning rate 5e-5, dropout rate 0.08 and number of base filters 16. The *in vivo* model was trained with the same parameters except with a dropout rate of 0.2 and number of base filters of 24. Model training required 2.5 days using four NVIDIA DGX A100 GPUs with 40 GB memory each.

### 2.5 Data Staging and Post-processing

Data staging consists of organizing, resampling, resizing, cropping and reformating MRI image volumes to be ready as input for data augmentation and network training, or for network inference. The software we developed, DeepBrainIPP, starts with a data staging step that significantly automates this process. Once the software is set up, users need only specify the location of an MRI dataset, and desired model type *via* the DeepBrainIPP interface (see Supplementary Figure S1 in Supplementary Material).

In addition, DeepBrainIPP performs post-segmentation processing to remove small fragments. They are detected and discarded by connected component analysis (Samet and Tamminen, 1988) and thresholding. Optionally holes in the output mask are filled. Total volumes of segmented brain and small regions are calculated for phenotyping.

### 2.6 Image Registration

#### 2.6.1 Atlas Selection and Brain Image Registration

Image registration is the process of aligning an image (the moving image) to a reference image (the fixed image) *via* a transformation model. If the reference image provides an associated set of labels to define substructures, then it is called an atlas. Atlas registration is therefore equivalent to image segmentation. One of the key factors that affect the quality of registration is the difference in modalities between the atlas and the moving image. We tested two widely used atlases in the literature (see Results) and decided to use a template from NeAt (Ma et al., 2005, 2008) for large area segmentation of *ex vivo* data (see rationale below). For *in vivo* images, we manually generated an in-house template based on the native modality of our data. NeAt’s template was downloaded from “https://github.com/dancebean/mouse-brain-atlas/tree/master/NeAt/ex_vivo/template” and selected by an expert. DeepBrainIPP, additionally, allows users to use their own templates if desired.

**FIGURE 7:**
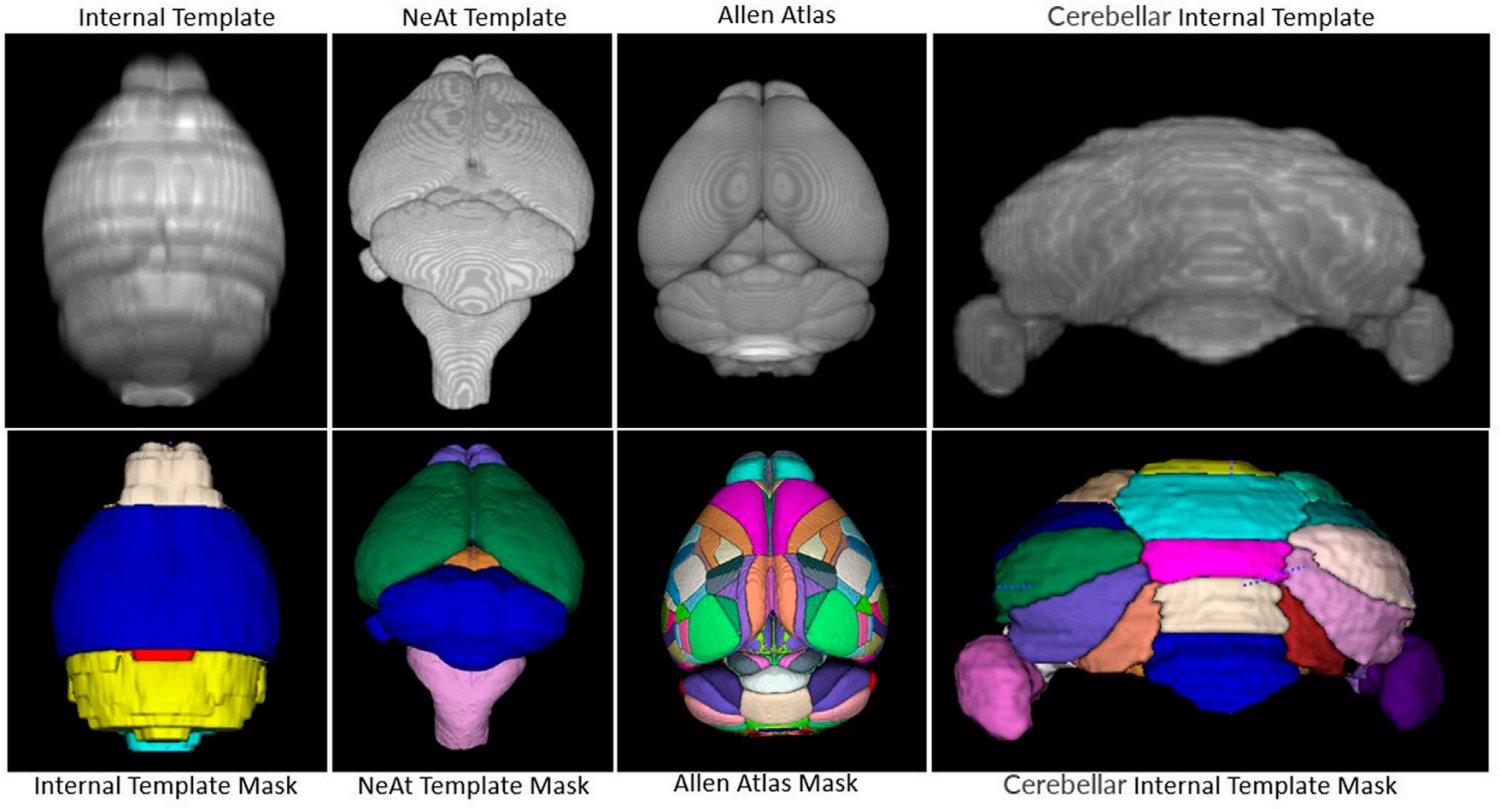
Atlas/templates: Top row shows atlas/templates and bottom row shows their mask. The whole brain internal template (top-left) and NeAt template (second from top-left) were used to register *in vivo* and *ex vivo* images respectively. The cerebellum internal template was developed to segment sub-cerebellar regions of *ex vivo* images. Brain structures are delineated with separate color in the mask.

**FIGURE 8:**
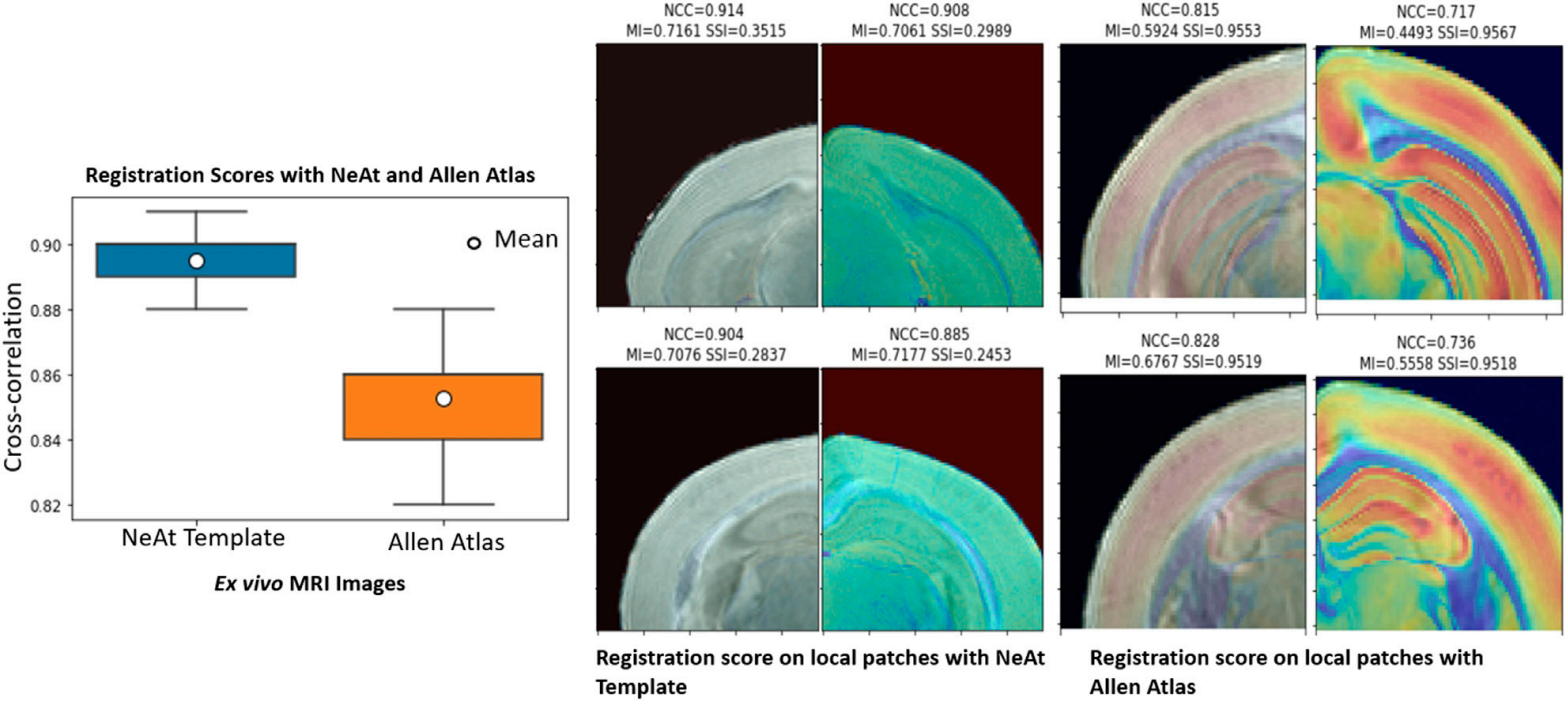
Atlas/template selection: Left panel: Registration scores of brain image with the NeAt templates and the Allen atlas. The overall registration score with NeAt template is better. Right panel: Similarity scores calculated from randomly selected local patches of a registered image with both reference images. Cropped patches are overlayed with corresponding atlas used for registration.

**FIGURE 9:**
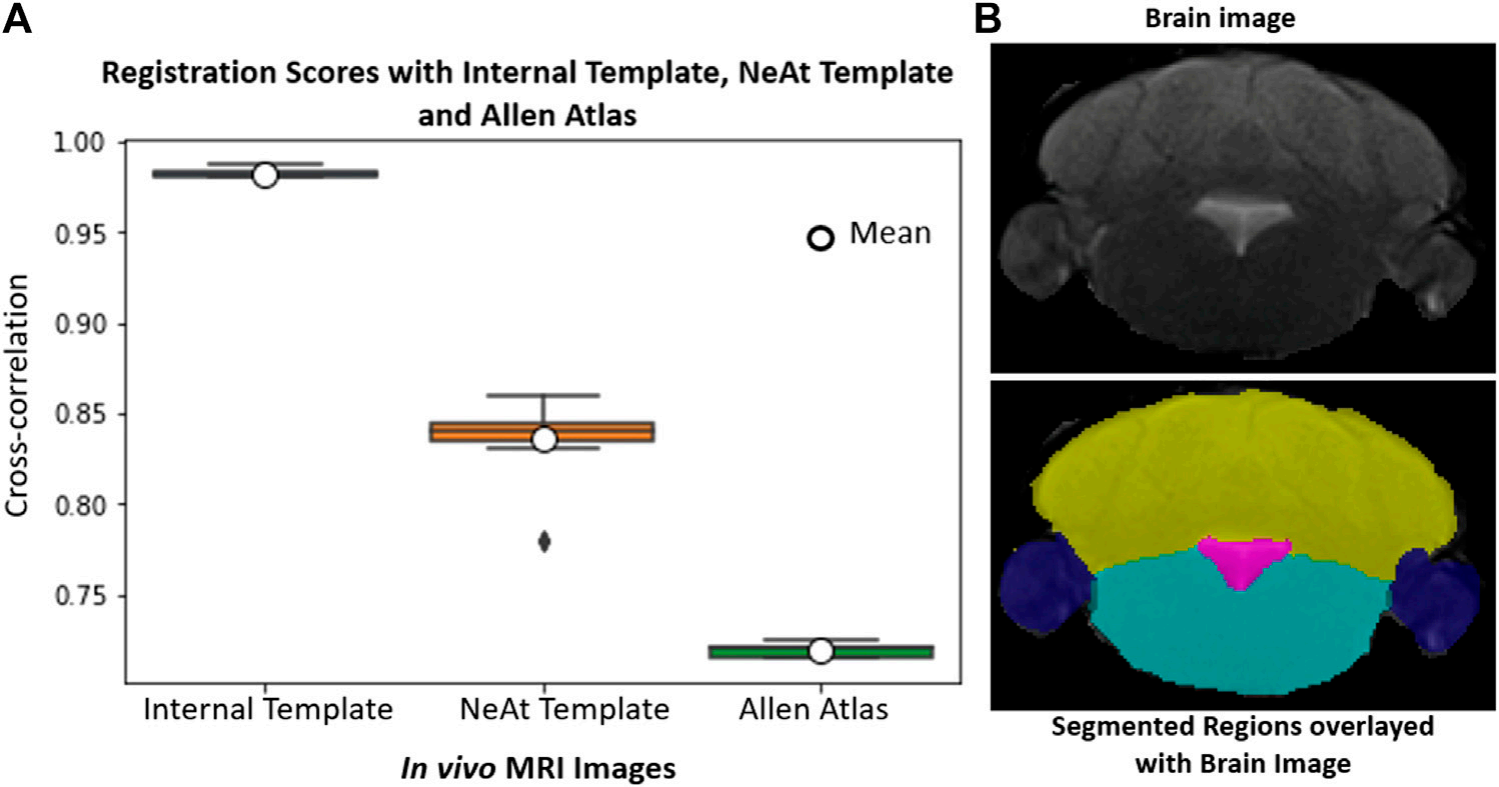
*In vivo* whole-brain registration: **(A)**: Registration scores of 18 image volumes with our internal template, NeAt template/Atlas and Allen atlas. **(B)**: Sample volume using the skull-stripping/registration paradigm with the internal (in-house) generated template and segmented brain structures. Each region is delineated with separate color; cerebellum (yellow), paraflocculus/flocculus (blue), ventricle (magenta), and brain stem (turquoise).

Our rationale for using the NeAt template, as opposed to the more widely used and more comprehensive Allen atlas: We compared registration quality by registering our skull-stripped brain volumes with NeAt (Ma et al., 2005, 2008), the Allen mouse brain atlas CCF-v3 (Wang et al., 2020) and an in-house developed template (see Figure 7). The Allen atlas contains hundreds of labeled structures and is widely used by researchers. However, NeAt contains a more detailed brain stem area, which is relevant to the biological studies that motivated this work and registers better to our data. We downsampled both the Allen atlas and NeAt templates to a resolution that closely matches our *in vivo* and *ex vivo* data to optimize registration time and accuracy. The Allen atlas was downsampled to 0.05 mm × 0.05 mm × 0.314 and 0.05 mm × 0.05 mm × 0.05 mm for *in vivo* and *ex vivo* respectively. The NeAt template was downsampled to 0.047 mm × 0.047 mm × 0.377 mm and 0.047 mm × 0.047 mm × 0.047 mm. We registered 14 *ex vivo* brain volumes with NeAt and the Allen atlas. In Figure 8 (left panel), we show registration scores for *ex vivo* data registered to the NeAt template and the Allen Atlas. The right panel of Figure 8 depicts the registration quality on local patches to examine how well internal structures align. Patches were randomly cropped from a registered volume and corresponding atlas, and then overlayed. Various similarity measures such as Normalized Cross-correlation (NCC), Mutual Information (MI), Structure Similarity Index (SSI) were calculated from local patches, and are shown in Figure 8. Volumes aligned better with the NeAt atlas compared to the Allen atlas both locally and globally. NeAt template’s better performance is likely due to the similarity in modality to our acquisitions (Brain MRI atlas of the wild-type C57BL/6J mouse strain). Based on these results, we selected the NeAt template as a reference image for *ex vivo* image registration. We found, however, that the registration quality for *in vivo* images was poor with both NeAt and the Allen atlas (see a comparion in Figure 9). Therefore, we developed an in-house template based on images native to our acquisition instruments. Once we concluded that our internal template works best, we registered (200+) *in vivo* images with it. The average registration score obtained from all MRI images is 0.93.

#### 2.6.2 Cerebellum Subregion Segmentation for *ex Vivo* Data

**FIGURE 10:**
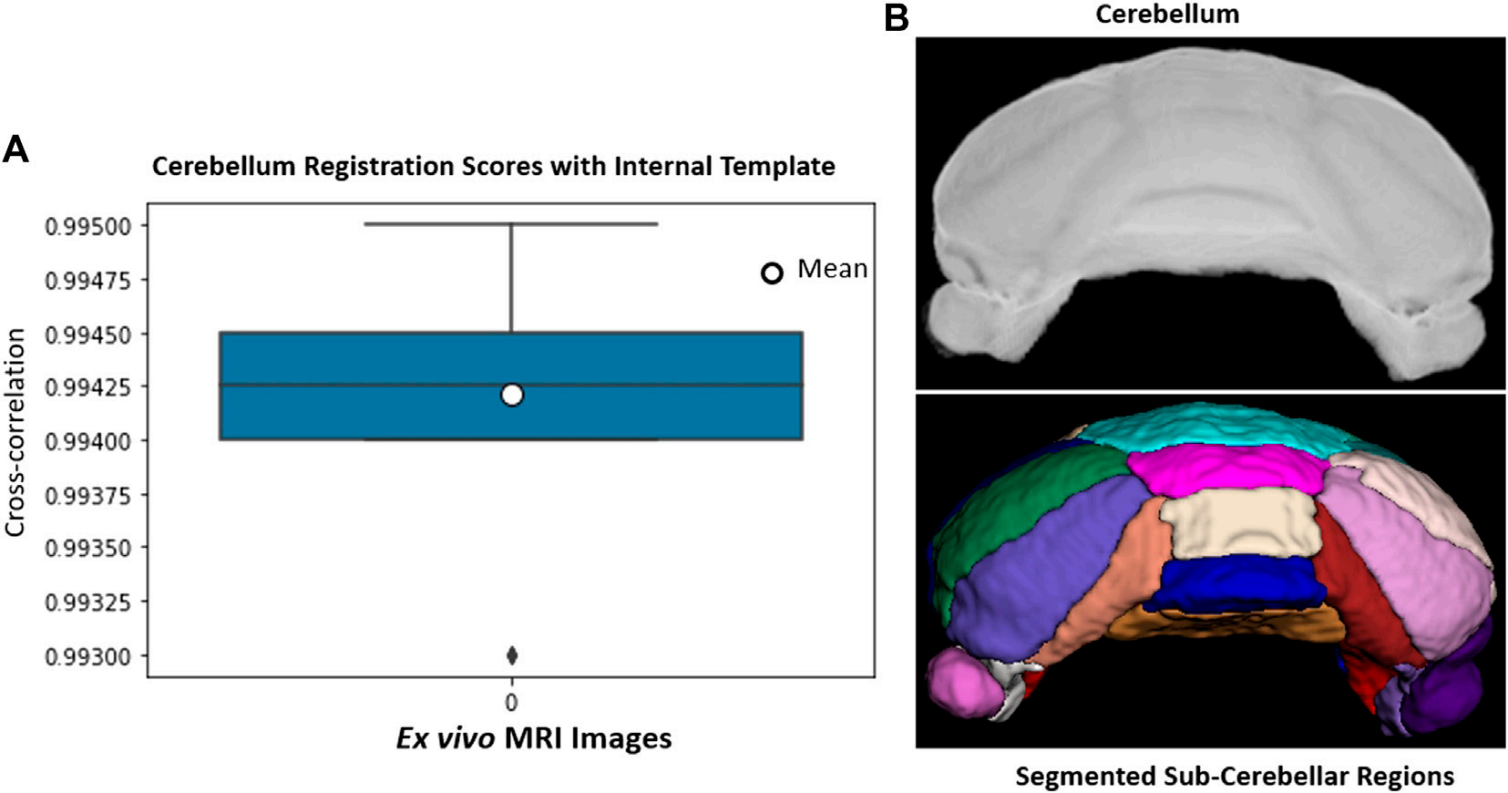
**(A)**: *Ex vivo* cerebellum registration: Registration scores for 14 cerebellum image volumes. **(B)**: A sample volume and segmented subcerebellar region surfaces. Each region is delineated with separate color; Vermis IV/V (cyan), Vermis VI (pink), Vermis VII (wheat), Vermis VIII (blue), Vermis IX (golden), Simple lobe (green and white), Crus I (navy, orchid), Crus II (salmon, maroon), Paraflocculus (purple, dark purple).

The cerebellum, obtained *via* skull-stripping followed by registration to the NeAT template, is registered to a manually generated in-house template that details subcerebellar regions. After registration, we apply inverse transformation to the template mask to extract the labeled subcerebellar regions in the original coordinate system of the input image. In Figure 10, we show registration scores and segmented subcerebellar regions for a sample volume. When subregions are very small and boundaries are not clear, segmentation accuracy is poor. For example, the average error in the measured volume of “Crus II” and “Paraflocculus” subregions are 20 and 9% respectively, well above the acceptable range of 6% (estimated human segmentation error bounds). To address this limitation, we segment smaller regions directly *via* deep learning.

#### 2.6.3 Registration Models and Parameter Selection

We use the state-of-the-art registration framework, ANTs (Avants et al., 2009) to register images. Registration was performed successively using a rigid transform, followed by affine and finally by a deformable model (Symmetric Normalization (Syn)) (Avants et al., 2008). Parameterization of the ANTs optimization process was based on a grid search considering image-contrast, volume, and image resolution. First, we find optimal parameters for a volume then use them to register entire batch (photometric properties and imaging condition remains same for a batch). Key parameters are summarized in Table 2. The entire set of ANTs parameters is exposed to the users *via* DeepBrainIPP’s interface (see “Interface for Image registration” in Supplementary Figure S1 in Supplementary Material).

**TABLE 2:** ANTs (Avants et al., 2009) transformation models and parameters used.

Transformation models	Parameter name	Parameter value/Range
Rigid	Gradient step size	0.1
	Number of Bins	(32,64,128)
	Metric	Mutual Information (MI)
	Shrink factor	(8,4,2,1)
	Smoothing Sigma	(8,6,4,1)
	Sampling Strategy	Regular
	Number of Levels	(1,2 4)
Affine	Gradient step size	0.1
	Number of Bins	(32,64,128)
	Metric	[Mutual Information (MI), Mattes]
	Shrink factor	(8,4,2,1)
	Smoothing Sigma	(8,6,4,1)
	Sampling Strategy	Regular
	Number of Levels	(1,2,4)
Syn (Symmetric Normalization)	gradient step size	0.1
	UpdateFieldVarianceInVoxelSpace	(3,7)
	TotalFieldVarianceInVoxelSpace	0
	Radius	(32,64,128)
	Metric	Cross Correlation (CC)
	Shrink factor	(8,4,2,1)
	Smoothing Sigma	(8,6,4,1)
	Sampling Strategy	Regular
	Number of Levels	(1,2,4)
	Interpolation	BSpline (3), Linear

### 2.7 Software

DeepBrainIPP is easily accessible *via* Web-browsers with a secure login. In order to use DeepBrainIPP, users do not need to install any packages and do not need to know the underlying algorithmic details for skull stripping, brain structure segmentation or morphology analysis.

DeepBrainIPP has one dependency; the image processing pipeline (IPP) which is available publicly (Janelia Research Campus, 2021), and must be set up once as a standing service by a systems administrator. The Image Processing Pipeline (IPP) is a service that allows users to run state-of-the-art image processing workflows on compute clusters *via* a web-based portal and convenient user interface. Different data processing steps—stored in a central code repository—can be strung together to create workflows of arbitrary complexity, with parameterization exposed to the user. Once a user has parameterized a workflow, they can submit it to run on a compute cluster (no scripting or coding skills needed). A dedicated database stores information about all compute jobs, including all user-set parameters and status. Past “runs” can be accessed *via* the front-end for easy reuse and monitoring facilitating data processing reproducibility.

**FIGURE 11:**
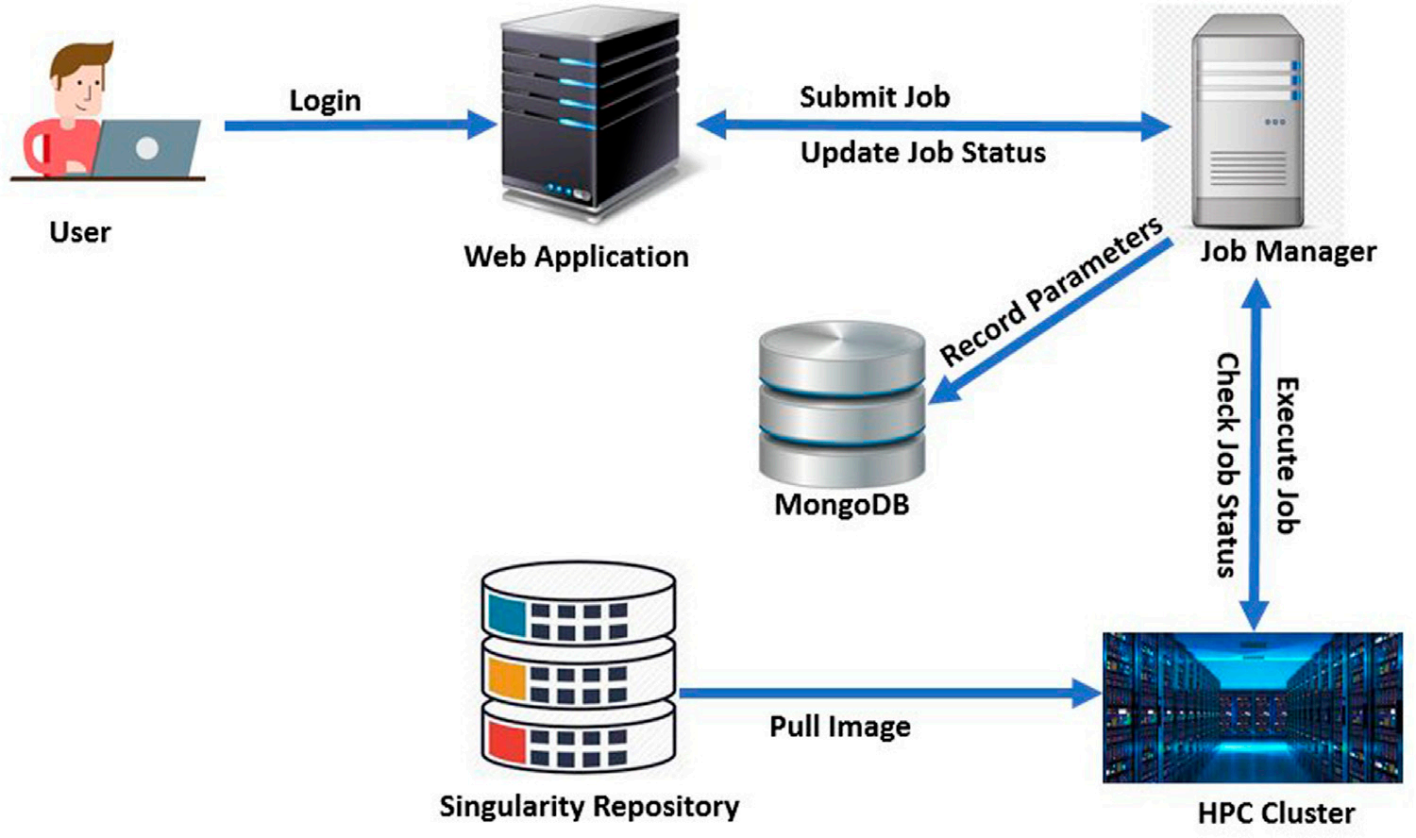
Architecture of DeepBrainIPP: DeepBrainIPP consists of four modules [web Application, job manager, singularity repository and High Performance Computing Cluster (HPC)].

DeepBrainIPP relies on the IPP to manage workflows and pipelines. We adapted and customized the (more general) IPP to build the final interface for DeepBrainIPP, and included neurobiologists and MRI specialists in the application development life cycle to understand issues related to usability and accessibility for this group. Moreover, we applied Design Thinking and System Thinking concepts to simplify the user-interface and to properly integrate components of DeepBrainIPP. The architecture of DeepBrainIPP is shown in Figure 11. The full application consists of four modules: 1. Web Application, 2. Singularity Repository 3. Job Manager, 4. High performance Compute (HPC) Unit. The web application handles user interaction *via* a web interface, which has two sections: “Admin” where administrators can create, configure and design workflows, and “User” for users to enter parameters and submit jobs. The user interface shows job status (“Created”,“Running”,“Successful”,“Error”) and information is updated in real-time. The user interface for skull stripping and image registration is shown in Supplementary Figure S1 (see Supplementary Material). In addition, the singularity repository associated with DeepBrainIPP contains all singularity-containers (each is a portable, reproducible and autonomous unit) and is accessible from HPC. We created a singularity-container for DeepBrainIPP by packaging all models, code, required libraries and dependencies into a single executable unit. The Job Manager receives job information, executes on HPC, monitors execution and updates the user. It also records parameters associated with each job in persistent storage (MongoDB) so that the full job can be reproduced in the future. The database has three replicated instances for disaster recovery and for sustained service availability.

## 3 Results and Discussion

### 3.1 Objective Evaluation

The quantitative evaluation of our deep neural network models was performed on independent test MRI images acquired with standard T2-weighted scans with two different coils (2-channel phased-array surface coil, 23 mm mouse head volume coil). The test set included image volumes of both mutant and wild-type mice, thereby spanning the relevant morphogenetic space for the studies that motivated this work. Below we describe our objective performance evaluation.

**FIGURE 12:**
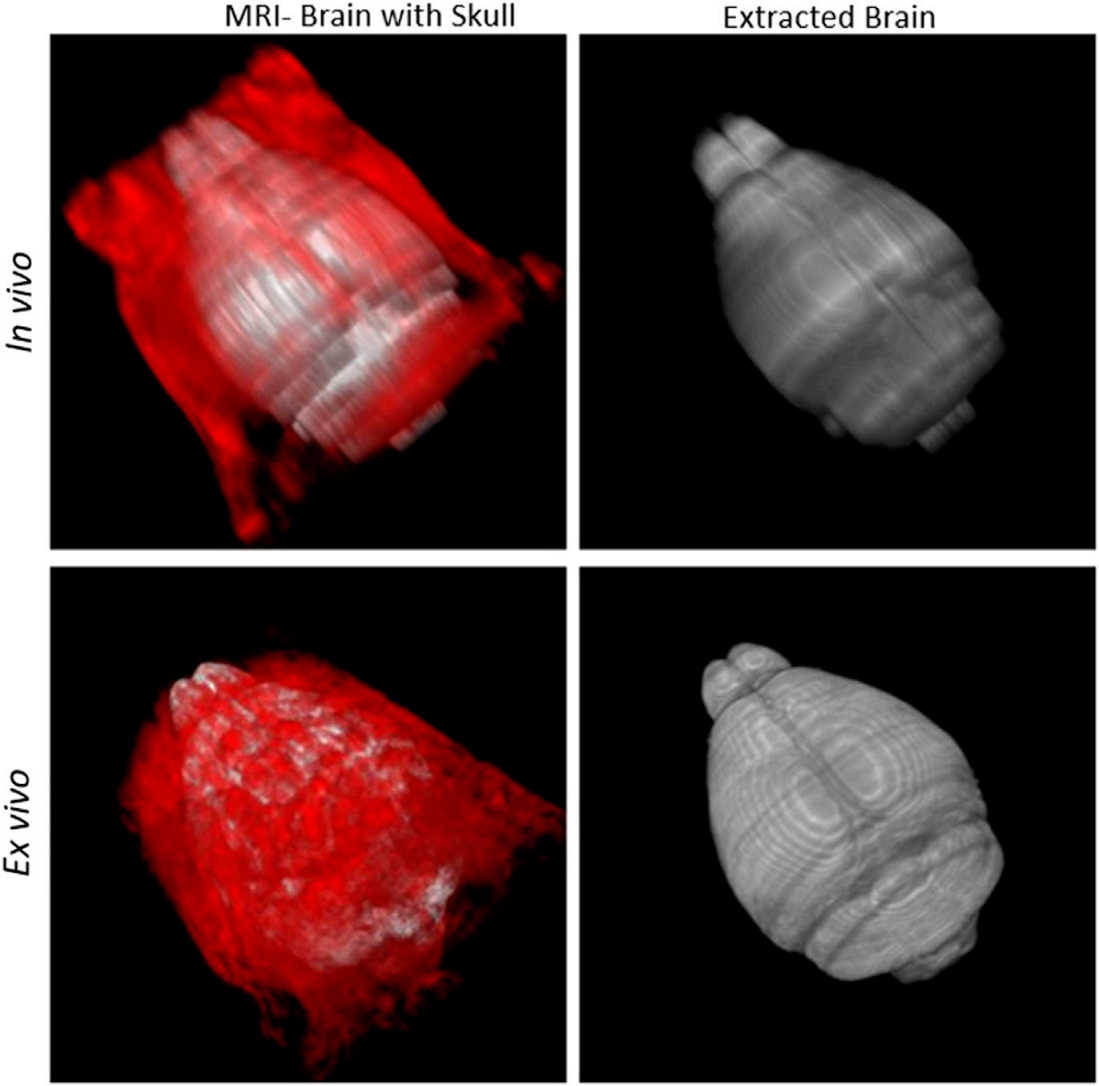
Skull-stripping: First column: Brain (grey) with skull (red). Second column: Segmented brain using DeepBrainIPP.

Segmentation outcomes were quantified using Dice score, Jaccard similarity, rate of true positive in prediction (PPV), sensitivity, and Hausdorff surface distance using Eqs 1–5 respectively. G and P represent voxels in ground truth and predicted mask, d (g, p) is the Euclidean distance between g and p. A volumetric similarity measure such as the Dice score is not sensitive to differences in edges or surface areas. Hence, we additionally use a Hausdorff surface distance metric to quantify the maximum contour distance between ground truth and the predicted masks. A low Hausdorff distance indicates edges or surface are well aligned. In Supplementary Table S2 (see Supplementary Material), we presented skull-stripping scores of our model along with two recently developed methods (Hsu et al., 2020; De Feo et al., 2021) as well as several state-of-the-art works. Hsu et al. (2020) applied several skull stripping methods such as U-Net (Hsu et al., 2020) et al., RATS (Oguz et al., 2014), PCNN (Chou et al., 2011), SHERM (Liu et al., 2020) on T2*w RARE images and summarized their segmentation performance (see Supplementary Table S2 in Supplementary Material). Our imaging was T2-weighted TSE with higher in-plane resolution but 0.5 mm thickness on a 7T MRI instrument which produces images similar to T2*w RARE images. Sample outcomes of skull stripping for both *in vivo* and *ex vivo* data using our models are shown in Figure 12.

$$Dice=2\left(\left|G\cap P\right|\right)/\left(\left|G|+|P\right|\right)$$ (1)

$$Jaccard=\left(\left|G\cap P\right|\right)/\left(\left|G\cup P\right|\right)$$ (2)

$$PPV=\left(\left|G\cap P\right|\right)/P$$ (3)

$$Sensitivity=\left(\left|G\cap P\right|\right)/G$$ (4)

$$\begin{array}{cc} Hausdorff & =max\left(h\left(G,P\right),h\left(P,G\right)\right)\\ h\left(G,P\right) & =max\left(min\left(d\left(g,p\right)\right)\right) \end{array}$$ (5)

Skull-stripped brains were registered with templates with an average cross-correlation of 0.98 and 0.89 for *in vivo* and *ex vivo* images respectively. From *ex vivo* and *in vivo* data, we segmented 20 and 11 large structures/regions of interest (see Supplementary Table S1 in Supplementary Material) by applying an inverse transformation to the template’s mask. The cerebellum, an example of a large structure, was segmented with an average volume residual of 4.1%. Segmented cerebellum images were registered with the internal template with an average cross-correlation 0.9942. We list 23 sub-cerebellar regions (see Supplementary Table S1 in Supplementary Material). This approach was successful for *ex vivo* data. Due to poor resolution and limited contrast, however, we do not segment sub-cerebellar regions of *in vivo* data *via* this template registration (It remains an option for users of DeepBrainIPP to proceed in this fashion). The paraflocculus was segmented directly yielding an average Dice score of 0.89, Jaccard similarity of 0.80, PPV of 0.89, sensitivity of 0.89, Hausdorff distance of 0.29 mm and volume residual of 5.32%.

We were inspired by MU-Net’s skull stripping performance reported and applied it to our dataset. However, MU-Net, with the original weights provided *via* the authors’ github repository at the time of this study, produced poor skull-stripping outcomes on the T2w TSE MRI images used in our study. We observed that the auxiliary bounding box detection network performs poorly on our data. Therefore, we manually cropped image volumes with a tight bounding box and used these images as input to MU-Net. The average Dice score obtained is 0.577, which does not meet our acceptance criteria and is significantly below the reported MU-Net (De Feo et al., 2021) performance. We investigated MU-Net’s generalizability, since the MRI training data used in the original MU-Net study were acquired with imaging parameters different than ours; significantly changing resolution and contrast when comparing the two datasets. MU-Net MRI image volumes were acquired using a TurboRARE sequence with effective TR/TE = 2,500/36 ms, matrix size 256 × 256, field of view 20.0 mm × 20.0 mm, 31 0.6 mm thick coronal slices, and a 0.15 mm inter-slice gap. Our training and TEST data (that most closely match the MU-Net training dataset) were captured using TR/TE = 2,500/42 ms, matrix size = 320 × 320, field of view = 25 mm × 25 mm, and a slice thickness of 0.5 mm. We therefore trained MU-Net network (SKULLNET: https://github.com/Hierakonpolis/MU-Net-R/blob/main/network.py) on our dataset, and it produced a segmentation score close to the one reported in the original MU-Net publication. Table 3 shows a comparison of skull stripping outcomes of DeepBrainIPP versus MU-Net on our dataset. Supplementary Figure S2 (see Supplementary Material) shows skull-stripping outcomes (predicted masks are shown in cyan color and overlaid with original volumes) on a sample volume produced by DeepBrainIPP and MU-Net (with and without retraining on our dataset). We note that MU-Net underestimates the boundary of the paraflocculus even after re-training on our dataset (see red box in Supplementary Figure S2C in Supplementary Material).

**TABLE 3:** Performance of skull stripping: DeepBrainIPP versus MU-Net.

Network*	Data augmentation scheme	Inference dataset	Dice	Jaccard	PPV	Sensitivity	Hausdorff	Residual volume (%)
DeepBrainIPP	No augmentation	in-house	0.94	0.90	0.96	0.92	0.78	4.3
DeepBrainIPP	With our augmentation	in-house	0.96	0.92	0.96	0.95	0.77	2.18
MU-Net (SKULLNET)	MU-Net’s augmentation	in-house	0.95	0.91	0.96	0.93	0.73	2.9
MU-Net (SKULLNET)	With our augmentation	in-house	0.95	0.91	0.96	0.94	0.79	1.55
DeepBrainIPP	No augmentation	NeAt	0.81	0.69	0.85	0.77	5.19	9.74
DeepBrainIPP	With our augmentation	NeAt	0.87	0.77	0.86	0.88	5.19	8.05

Network*:All these networks were trained on our in-house dataset.

In-house**:T2*W TSE, with higher in-plane resolution.

We also investigated the power of our trained model to generalize to the externally published NeAt dataset (MRI images differ in resolution and contrast with our training samples). Without fine-tuning, our model was able to skull strip NeAt data with an average Dice score of 0.92 for 6/10 volumes. For the remaining four volumes, which are more extreme scans, the Dice score was unacceptable at 0.80, likely requiring fine tuning (see sample skull stripping outcomes of DeepBrainIPP on NeAt data in Supplementary Figure S3 in Supplementary Material).

We therefore conclude that the skull stripping model developed in this work performed better than (or comparable to) state-of-the-art models on T2w TSE mouse MRI data. The key reasons are: 1) Our model was trained with an extensive augmentation scheme, possibly with samples that represent more comprehensively potential input data. Our data augmentation scheme increased the segmentation score (dice) of DeepBrainIPP by 7.4% on the NeAt dataset. It also increased the skull stripping outcome and reduced error in measured volume when used to train MU-Net [see Table 3) 2] We performed an extensive hyperparameter optimization to choose the best performing network architecture. 3) The network architecture contains residual units (He et al., 2016) that enable training very deep networks without suffering from vanishing gradients and provide the potential to learn complex data relationships (Kayalibay et al., 2017).

### 3.2 Limitations

The performance of our skull-stripping procedure degraded noticeably in cases of severe hydrocephalus in the brain. The excess fluid present in the cavity (hydrocephalus) decreases contrast of boundaries rendering the segmentation task more challenging. Our approach to segment small brain regions directly, requires an extra step of training the model each time when a new region is added to the list. The reason is when a new region is added the training dataset changes, which triggers a need to re-estimate optimal hyperparameter values.

### 3.3 Subjective Evaluation

We conducted a brief study for the subjective evaluation of DeepBrainIPP by its intended users. The key purpose of subjective evaluation of an application is to gather user experience (Hassenzahl and Tractinsky, 2006) which requires a carefully crafted questionnaire. Koumpouros and others (Koumpouros, 2016) recommended two scales, QUEST 2.0 and PYTHEIA for the subjective evaluation of a system from a comprehensive study. QUEST is very generic and was not used widely. PYTHEIA scale is used to measure reliability and validity of assistive services (Anam et al., 2014; Alam et al., 2016; Ahmed et al., 2018; Alam et al., 2020; Alam, 2021). However, it can be customized to evaluate other applications as well. We prepared a questionnaire consisting of 17 statements/questions following PYTHEIA specifications (see Supplementary Figures S4, S5 in Supplementary Material) and invited nine users (3 image analysts, one cell and molecular biologist, one preclinical imaging analyst, two neurobiologists, two computational engineers) to participate in a user study. Participants rated their satisfaction with DeepBrainIPP in a 6-point Likert-scale. A subset of participants have been using DeepBrainIPP since August 2021. To ensure the reliability of the Likert-scale survey, PYTHEIA measures: 1) internal consistency-evaluates how well different questions (items) that test the latent structure of the system give consistent results; 2) test-retest reliability-evaluates the degree to which participants maintain their opinion in repeated experiments 3) repeatability-measures consistency of a system’s outcome whenever it is used and the stability of a user’s opinion. Internal consistency, test-retest reliability, and repeatability were measured with Cronbach’s alpha, intra-class correlation coefficient (ICC), and Pearson’s product-moment correlation coefficient respectively. The measured Cronbach’s alpha is 0.930 (see detail in Supplementary Table S3 in Supplementary Material), shows sufficient consistency among the statements (*α*, 0.0 = “no consistency”, 1.0 = “perfect consistency”, greater than 0.7 = “sufficient consistency”) which indicates various features of DeepBrainIPP received consistence score from the participants. The test-retest reliability is 0.816, indicating stability of the system’s performance whenever it was used. The Pearson coefficient is 0.7, showing that participants maintained moderate consistency in their opinion. The questions/items received an average score of 5.3 (see details score in Supplementary Table S4 in Supplementary Material) and participants provided an average score of 5.3 (see details score in Supplementary Table S5 in Supplementary Material) to a question, which indicates users were satisfied with DeepBrainIPP. The measured Customer Satisfaction Score (CSAT) is 89%. CSAT score was calculated from the ratio of the total number of customers who rated four or above and the total number of participants.

Although, participants were satisfied with DeepBrainIPP they made some feature requests. For example, one of the participants asked to include a light 3D visualization tool in DeepBrainIPP so that they can explore segmentation outcomes *via* a Web interface. Another participant asked to make the analysis pipeline accessible to external users. For external users, we have made the code-base, models and other components available in https://github.com/stjude/DeepBrainIPP with detailed instructions on how to set it up and use it. In addition, we have a future plan to deploy DeepBrainIPP on Amazon AWS so that it becomes even more accessible. Another participant asked to read the metadata such as voxel spacing from MRI headers during automatic file organization to save entering metadata in the web-form (see “Interface for Skull Stripping” in Supplementary Figure S1 in the Supplementary Material). We have found several cases where imaging instruments did not export metadata properly. Hence, we encourage users to supply that information during job submission. We plan for DeepBrainIPP to automatically check any metadata inconsistency (between user-entered inputs and file headers) and report discrepancies to the user before job submission.

## 4 Conclusion

In this paper, we presented DeepBrainIPP, an integrated end-to-end solution for automated brain structure segmentation. Our approach addresses several research and technological challenges in the context of MRI mouse brain image analysis; development of a robust fully automated model for skull stripping and segmentation of mouse brain structures, development of data augmentation strategies that counter the small annotated dataset size, and development of a scalable pipeline that is accessible to non-computational research staff. The software is modular and therefore allows additional brain regions to be integrated into the existing workflow. DeepBrainIPP produced segmentation outcomes at a performance level of the human annotator. However, we have not tested how well our models generalize in context of similar data acquired by different instruments or for non-brain recordings.

## Data Availability

The data analyzed in this study is subject to the following licenses/restrictions: Full image data will be released as part of a separate study. Code, network weights and sample image volumes are made available as part of the code release. Requests to access these datasets should be directed to https://github.com/stjude/DeepBrainIPP.
