# Supplementary material for: An End-To-End Pipeline for Fully Automatic Morphological Quantification of Mouse Brain Structures From MRI Imagery
Source: Front Bioinform. 2022 Jun 8;2:865443. doi: 10.3389/fbinf.2022.865443 (PMC9580949; doi:10.3389/fbinf.2022.865443)
Supplement: Supplementary file 1 [file DataSheet1.pdf]

Supplementary Material for "An end-to-end pipeline for fully automatic morphological quantification of mouse brain structures from MRI imagery"

1 MODEL PARAMETERS AND MEMORY REQUIREMENTS

The network architecture of DeepBrainIPP is deep, and we trained the model on the full image. therefore, it required a large amount of GPU memory to store outcomes of intermediate layers. The memory requirement below is only approximate due to additional memory needs for the Adam optimizer and implementation overhead. The ex vivo models have 8,268,743 trainable parameters, with an estimated memory requirement of 32 MB. The required memory for storing intermediate layer outputs for input dimensions of 256 x 224 x 288 was approximately 32,513MB. The additional memory required for the Adam optimizer and implementation was approximately 5596MB. Therefore, the total required memory to train ex vivo models was approximately 38 GB with a batch size of 1. Similarly, the total required memory for in vivo models with an input dimension of 448 x 448 x48, and trainable parameters of 8,273,867 is approximately 23 GB for a batch size of 1. Given limitations of GPU memory available for this study, training was restricted to a batch size of one.

2 SUPPLEMENTARY TABLES AND FIGURES

Table S1. Brain structures segmented from ex vivo and in vivo MRI image volumes

Segmented structures (ex vivo)	Sub-Cerebellar structures (ex vivo)	Segmented structures (in vivo)
Hippocampus	Vermis-I-II	Cerebellum
External-Capsule	Vermis-IV-V	External-Capsule
Caudate-Putamen	Vermis-VI	Hippocampus
Globus-Pallidus	Vermis-VII	Brain-stem
Thalamus	Vermis-VIII	Ventricles
Superior-Colliculi	Vermis-IX	lfactory-bulb
Ventricles	Vermis-X	Cortex
Hypothalamus	Simple-Lobule–left	Hypothalamus
Inferior-Colliculi	Simple-Lobule-right	Thalamus
Central-Gray	Crus-I–left	Caudate-Putamen
Anterior-Commissure	Cerebellum	Rest-of-Brain
Cerebellum	Crus-I-right	
Internal-Capsule	Crus-II–left	
Neocortex	Crus-II-right	
Amygdala	Paramedian-Lobule–left	
Olfactory-bulb	Paramedian-Lobule-right	
Brain-Stem	Copula-left	
Rest-of-Midbrain	Copula-right	
Basal-Forebrain-Septum	Paraflocculus-left	
Fimbria	Paraflocculus-right	
	Flocculus-left	
	Flocculus-right	
	Arbor-Vitae	

Table S2. Performance of Skull Stripping of DeepBrainIPP and State-of-the-art methods on various dataset

Network	TEST Dataset (mouse MRI images)	Dice	Jaccard	PPV	Sensitivity	Hausdorff
DeepBrainIPP	T2w TSE	**0.96**	**0.92**	**0.96**	**0.95**	**0.77**
MU-Net	T2w	0.97*	-	0.96	-	-
U-net	CAMRI dataset (T2w RARE)	0.85	0.74	0.74	0.98	5.23
RATS	CAMRI dataset (T2w RARE)	0.82	0.70	0.76	0.91	5.07
PCNN	CAMRI dataset (T2w RARE)	0.79	0.65	0.76	0.83	7.07
SHERM	CAMRI dataset (T2w RARE)	0.80	0.67	0.72	0.90	7.03

*We were not able to reproduce this Dice score for our dataset using published network weights.

Table S3. Item/Question PYTHEIA Analysis.

Questions/Items	Scale Mean if Item Deleted	Scale Variance if Item Deleted	Corrected Item-Total Correlation	Cronbach's Alpha if item Deleted
Question1	85.00	92.250	0.878.	0.921
Question2	85.67	97.750	0.582.	0.928
Question3	85.44	95.778	0.565.	0.928
Question4	85.22	90.444	0.840.	0.921
Question5	85.33	101.000	0.267.	0.933
Question6	85.56	89.528	0.604.	0.929
Question7	85.11	92.361	0.869.	0.922
Question8	85.11	86.361	0.936.	0.918
Question9	85.44	83.778	0.845.	0.921
Question10	85.22	86.944	0.916.	0.918
Question11	84.89	106.611	-0.143	0.941
Question12	84.89	101.361	0.222.	0.934
Question13	85.33	92.250	0.634.	0.926
Question14	85.56	94.278	0.502.	0.930
Question15	85.00	91.750	0.916.	0.921
Question16	85.22	87.694	0.872.	0.920
Question17	84.89	98.361	.647.	.927

Table S4. The average score received by each item/question from all participants.

Questions/Items	Mean	Std. Deviation	N (Number of Participants)
Question1	5.56	0.726	9
Question2	4.89	0.601	9
Question3	5.11	0.782	9
Question4	5.33	0.866	9
Question5	5.22	0.667	9
Question6	5.00	1.225	9
Question7	5.44	0.726	9
Question8	5.44	1.014	9
Question9	5.11	1.269	9
Question10	5.33	1.000	9
Question11	5.67	0.707	9
Question12	5.67	0.707	9
Question13	5.22	0.972	9
Question14	5.00	1.000	9
Question15	5.56	0.726	9
Question16	5.33	1.000	9
Question17	5.67	0.500	9

Table S5. The average score provided by participants.

Participants	Mean	Std. Deviation	N (number of statements)
Participant1	5.35	0.493	17
Participant2	5.59	0.507	17
Participant3	5.71	0.588	17
Participant4	5.47	0.624	17
Participant5	5.82	0.529	17
Participant6	5.41	1.004	17
Participant7	3.94	1.088	17
Participant8	5.82	0.393	17
Participant9	4.82	0.393	17

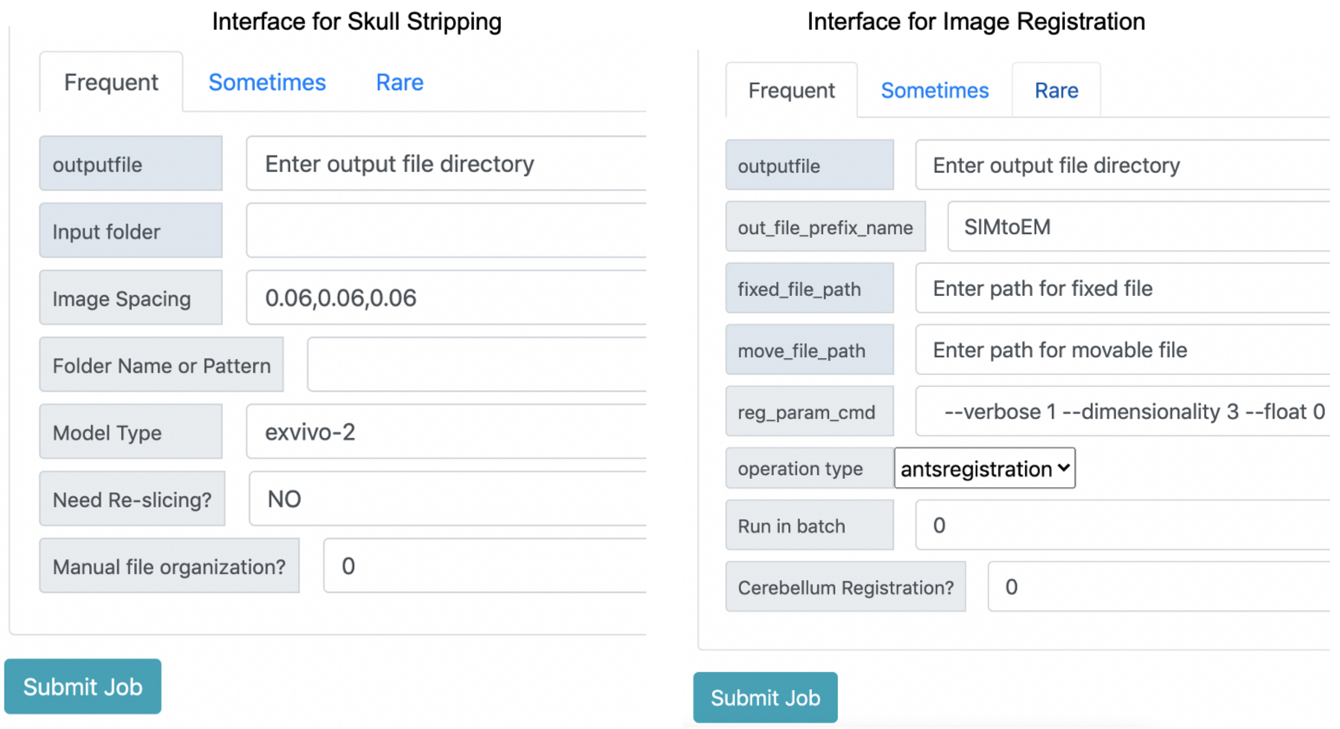

Figure S1: Web interface screenshot: Left: Skull stripping. Right: Image registration. DeepBrainIPP automatically organizes files and allows users to batch process.

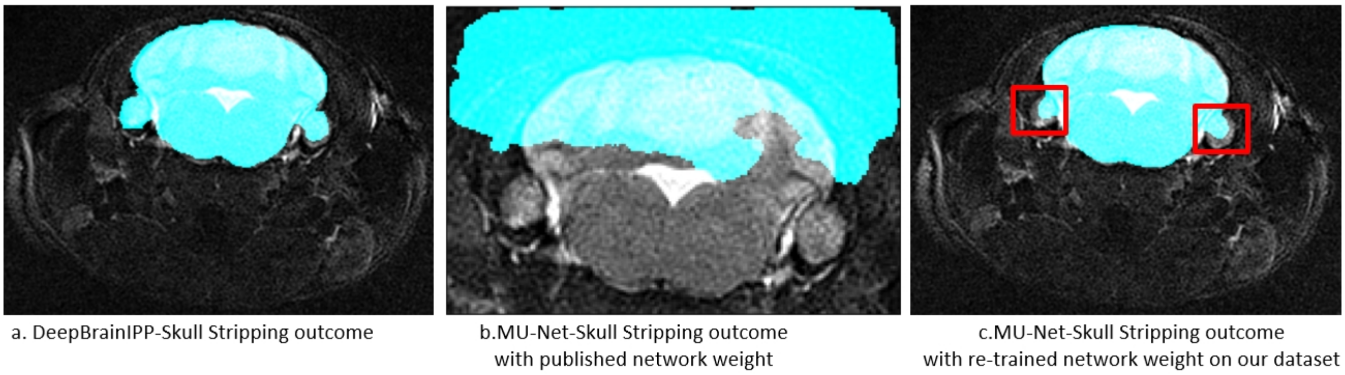

Figure S2: Skull stripping comparison: DeepBrainIPP vs MU-Net. (a) DeepBrainIPP skull-stripping captures the large-scale outline and paraflocculus lobules well. (b) MU-Net (published weights) underestimates and overestimated brain regions. (c) MU-Net(SKULLNET), trained on our dataset and with our data augmentation, significantly improves skull stripping, although paraflocculus was not segmented accurately (red box). Prediction masks: cyan overlayed with original data.

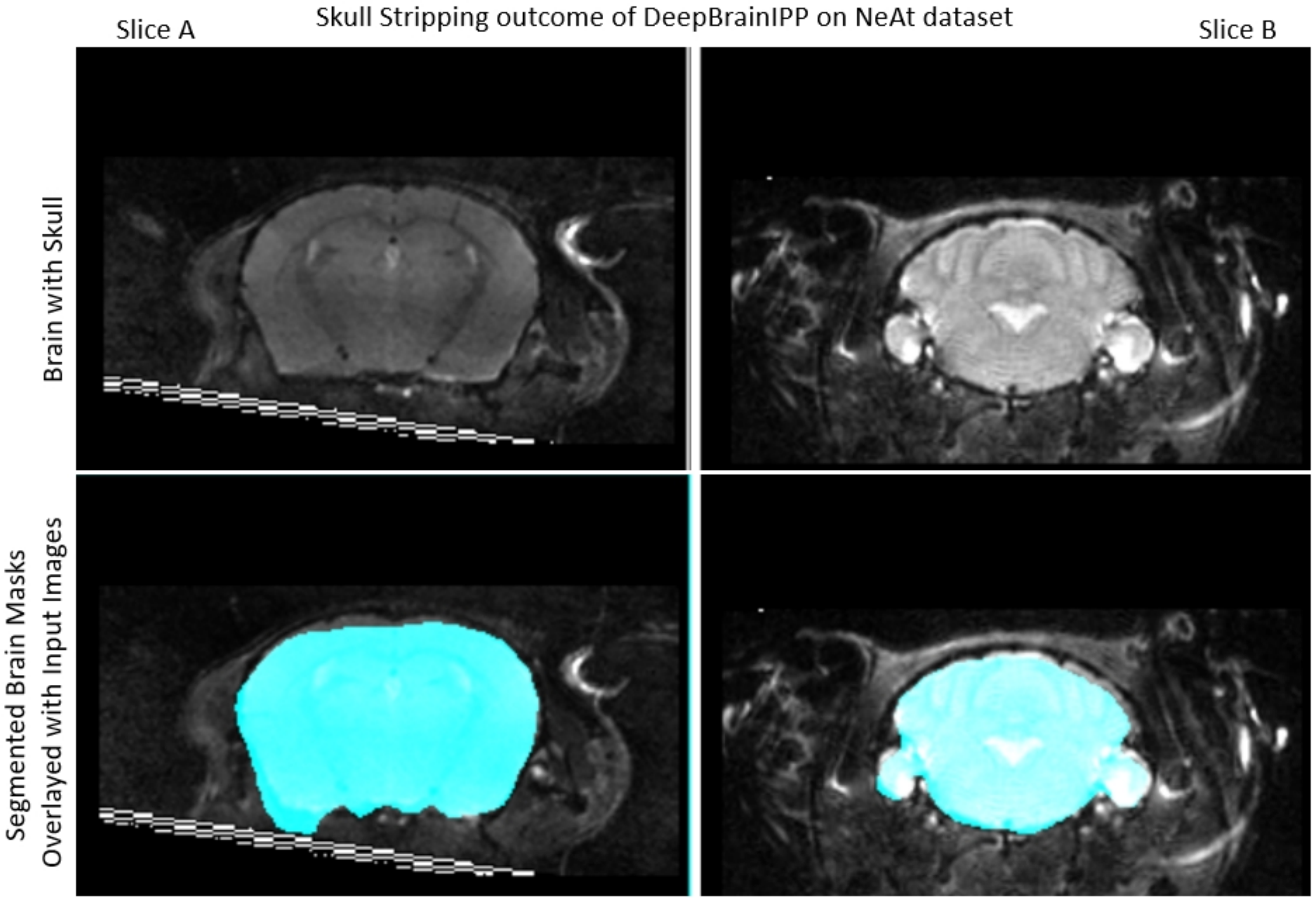

Figure S3: Example outcome of DeepBrainIPP trained on our in-house dataset when applied to skull-stripping segmentation of the NeAt dataset. Prediction masks (cyan color) overlayed with original data. Slice A and Slice B are random z-slices captured from a sample volume.

Questionnaire for user study

Participants Name: Area of Expertise:

The following statements are to evaluate effectiveness of DeepBrainIPP as a whole. Please rate your satisfaction with, and confidence in, the following statements on a 6-point scale.

Likert scale for question 1-10

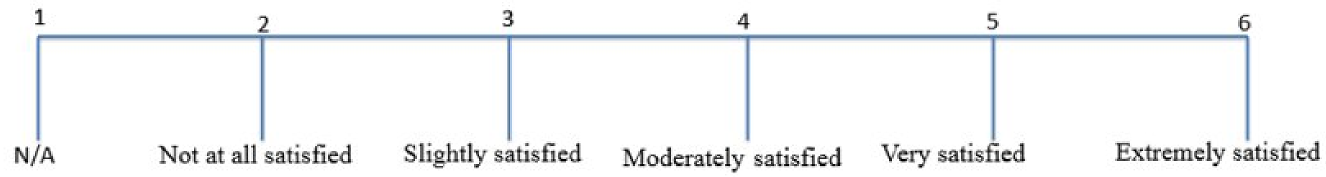

Likert scale for question 11-18

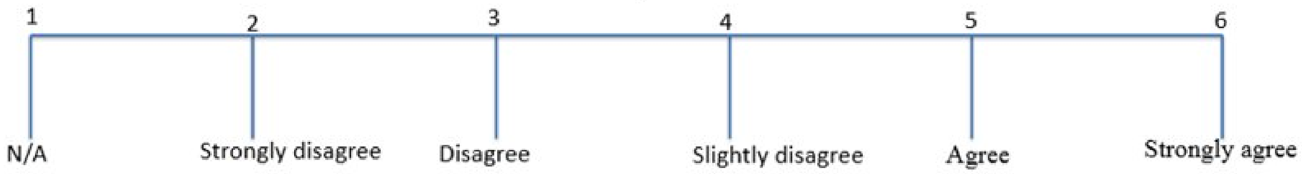

1. Rate your satisfaction with DeepBrainIPP in relation to its accessibility through a web browser	
2. Rate your satisfaction with DeepBrainIPP in relation to the ease of learning all individual functions.	
3. Rate your satisfaction with DeepBrainIPP in relation to the ease of use (complexity, required effort).	
4. Rate your satisfaction with DeepBrainIPP in relation to sufficiency of existing functions	
5. Rate your satisfaction with DeepBrainIPP in relation to ease of job submissions, management, and monitoring	
6. Rate your satisfaction with DeepBrainIPP in relation to ease of entering/modifying parameters	
7. Rate your satisfaction with DeepBrainIPP to segment mouse brain data	
8. Rate your satisfaction with DeepBrainIPP to register an extracted brain to an atlas and segment larger regions	
9. Rate your satisfaction with DeepBrainIPP to segment smaller brain regions	
10. Rate your satisfaction with DeepBrainIPP in relation to its effectiveness as a tool for mouse brain structure segmentation	

Figure S4: Questionnaire for subjective evaluation and to measure user satisfaction.

The following statements are to evaluate the effectiveness of utility features of DeepBrainIPP.

11. I do not need to install any software/packages/dependencies to use DeepBrainIPP	
12. I do not need to know details of the image analysis algorithms to segment mouse brain structures	
13. I don't need to perform any special dataset organization or preprocessing	
14. DeepBrainIPP produces results sufficient for morphogenetic characterization	
15. I feel more autonomous when I use DeepBrainIPP for mouse brain structures segmentation	
16. DeepBrainIPP eliminates the burden of manual segmentation	
17. I will recommend DeepBrainIPP to others	

Comments/Suggestions:

Figure S5: Questionnaire for subjective evaluation and to measure user satisfaction.
